# Impact of Temperature Optimization of ITO Thin Film on Tandem Solar Cell Efficiency

**DOI:** 10.3390/ma17112784

**Published:** 2024-06-06

**Authors:** Elif Damgaci, Emre Kartal, Furkan Gucluer, Ayse Seyhan, Yuksel Kaplan

**Affiliations:** 1 Department of Mechanical Engineering, Nigde Omer Halisdemir University, Nigde 51240, Türkiye; ykaplan@ohu.edu.tr; 2 Nanotechnology Application and Research Center, Nigde Omer Halisdemir University, Nigde 51240, Türkiye; emre-kartal@mail.ohu.edu.tr (E.K.); furkangucluer@hotmail.com (F.G.); aseyhan@ohu.edu.tr (A.S.); 3 Department of Physics, Nigde Omer Halisdemir University, Nigde 51240, Türkiye

**Keywords:** indium tin oxide (ITO), tandem solar cell, silicon heterojunction, perovskite, deposition temperature

## Abstract

This study examined the impact of temperature optimization on indium tin oxide (ITO) films in monolithic HJT/perovskite tandem solar cells. ITO films were deposited using magnetron sputtering at temperatures ranging from room temperature (25 °C) to 250 °C. The sputtering target was ITO, with a mass ratio of In_2_O_3_ to SnO_2_ of 90% to 10%. The effects of temperature on the ITO film were analyzed using X-ray diffraction (XRD), spectroscopic ellipsometry, and sheet resistance measurements. Results showed that all ITO films exhibited a polycrystalline morphology, with diffraction peaks corresponding to planes (211), (222), (400), (440), and (622), indicating a cubic bixbyite crystal structure. The light transmittance exceeded 80%, and the sheet resistance was 75.1 Ω/sq for ITO deposited at 200 °C. The optical bandgap of deposited ITO films ranged between 3.90 eV and 3.93 eV. Structural and morphological characterization of the perovskite solar cell was performed using XRD and FE-SEM. Tandem solar cell performance was evaluated by analyzing current density-voltage characteristics under simulated sunlight. By optimizing the ITO deposition temperature, the tandem cell achieved a power conversion efficiency (PCE) of 16.74%, resulting in enhanced tandem cell efficiency.

## 1. Introduction

The global photovoltaic (PV) market is predominantly led by crystalline silicon (c-Si) solar cells, which constitute over 95% of the market share, however, in the dynamic of the PV sector, new photovoltaic materials are continually emerging, with perovskite standing out as a promising alternative to Si-based solar cells for its versatile applications in solar cells, LEDs, and even electrochemical water splitting, showcasing its growing importance in optoelectronics and beyond [1,2]. Recent advancements in perovskite solar cells (PSCs), with an efficiency ranging from 3.13% to 26.1% between 2009 and 2023, underscore their potential as a cutting-edge solar technology [3]. The Shockley–Queisser limit represents the maximum theoretical efficiency that a single-junction solar cell can achieve based on thermodynamic principles which is about 33.7% for a single-junction solar cell under standard test conditions; it is determined via the balance between photon absorption and thermalization losses. Integrating perovskites with crystalline silicon (c-Si) solar cells offers significant promise, surpassing the Shockley–Queisser limit for single-junction solar cells [4] and boosting power conversion efficiency (PCE), thereby driving down the cost of electricity in photovoltaic systems. Jacak et al. also emphasized the inherent limitations of p-n junction solar cells, defined by the Shockley–Queisser limit. At the same time, they highlighted the innovative potential of perovskite solar cells to overcome these limitations. Third-generation cells, such as perovskite cells, achieve efficiencies comparable to p-n junction cells. Furthermore, they can enhance operational efficiency by up to 40% through the use of low-cost metallization, low-temperature processing, and simple manufacturing techniques, rendering them a competitive solution for future large-scale applications [5,6].

The c-Si HJT/perovskite tandem structure merges superior optoelectronic properties of perovskites with the high short circuit current of c-Si HJT cells, making it ideal for photovoltaic devices [7,8]. While solution-based perovskite cell processes offer cost-effectiveness and easy manufacturability, they face challenges such as degradation [9,10,11,12]. The c-Si subcell addresses degradation concerns with its high environmental and thermal stability, alongside compatibility with industrial workflows and the use of n-type silicon wafers for prolonged lifespan and reduced degradation. These advancements, coupled with increased efficiency and affordability, position the c-Si HJT/perovskite tandem structure as a promising contender for future photovoltaic technology phases, leveraging advantages like improved surface passivation and a favorable temperature coefficient [13]. The fabrication of the top cell layers in a monolithic tandem stack is restricted by the properties of the bottom cell, including required temperature, utilized solvents, and deposition technique. This can complicate tandem integration, which involves all required processing steps in the monolithic stack that may interfere with each other [7]. Transparent conductive oxides (TCOs) play a crucial role in HJT solar cells, serving as transparent electrodes that allow sunlight to reach the active layer while providing a conductive pathway for generated electricity, leading to enhanced efficiencies [14,15,16,17]. ITO combines high electrical conductivity and optical transparency, particularly in the visible spectrum. It has high hardness, wear resistance, and chemical corrosion resistance, ensuring durability. Limited indium sources, brittleness, and high raw material costs are challenges associated with ITO [18]. Conversely, when compared to other TCO materials, doped zinc oxide (ZnO) exhibits properties similar to those of ITO in terms of both band gap and optical transmission values. Aluminum-doped zinc oxide (AZO) has recently attracted significant attention for its cost-effectiveness and high electrical conductivity. Additionally, its nontoxicity and excellent transmittance further enhance its appeal [19]. Nevertheless, a downside of AZO thin films is their high absorption in the infrared (IR) region of the electromagnetic spectrum, which consequently diminishes the photo-generated current in solar cells. This absorption behavior is a crucial factor to consider in optimizing the performance of solar cell technologies [20]. ITO:Zr films, in comparison to ITO films, demonstrate superior electrical and optical properties. The addition of Zr to ITO films preserves the basic characteristics of ITO while also increasing properties such as near-infrared (NIR) transmittance, chemical stability, and thermal stability making them promising for future solar cell applications [21]. Indium tungsten oxide (IWO) and indium chromium oxide (ICO) films, similar to ITO, are transparent conducting oxide (TCO) materials, though less commonly used due to various factors. IWO and ICO films exhibit reasonable electrical conductivity, moderate to high optical transparency, and good durability. However, matching their electrical and optical properties to specific applications can be challenging due to their less predictable behavior. Additionally, large-scale industrial production of both IWO and ICO films poses difficulties compared to more established TCO materials like ITO [22,23]. Ti-doped In_2_O_3_ exhibits higher mobility and optical transparency compared to ITO thin films, especially in the visible light spectrum. However, a high-temperature process is often preferred to enhance the mobility of these materials. This situation is not suitable for optoelectronic applications requiring low temperatures, such as SHJ solar cells [24]. ITO plays a crucial role in tandem solar cells. Its high optical transparency ensures that a significant portion of incident sunlight can reach the underlying solar cell layers without substantial absorption or reflection, maximizing light absorption and overall tandem cell efficiency. ITO provides efficient charge collection by facilitating the transport of electrons from the tandem solar cells to the external circuit. Its high electrical conductivity allows for low resistive losses and efficient extraction of generated electrical current. Mesmer et al. simulated ITO thin film deposition at around 200 °C, achieving a c-Si/perovskite tandem solar cell efficiency of approximately 29.5% in a 5-busbar cell with an oxygen flow rate of 3 sccm [25]. Ba et al. explored tungsten-doped tin oxide, a less common TCO, in the conventional HJT subcell structure of a tandem cell, achieving approximately 23% efficiency for M_0_ (156 mm × 156 mm) size cells in their simulation study [26]. In summary, ITO serves as a crucial component in tandem solar cells by providing efficient light transmission, charge collection, and interface engineering, contributing to enhanced cell efficiency and performance [27]. The electrical conductivity and optical transparency of ITO films are significantly influenced by the deposition temperature. Higher deposition temperatures often result in higher conductivity due to improved crystallinity and reduced defects. Deposition temperature affects the grain size and crystallinity of ITO films. Generally, films deposited at higher temperatures exhibit larger grains and improved crystallinity, leading to smoother and more uniform films. The optical transparency of ITO films can vary with deposition temperature. Films deposited at specific temperatures often show higher transparency in the visible spectrum due to reduced grain size and crystallinity. There is typically a trade-off between transparency and conductivity, with films deposited at higher temperatures having lower transparency but higher conductivity, and vice versa. The stability of ITO films under different environmental conditions, such as humidity and temperature fluctuations, varies depending on the deposition temperature. Therefore, it is important to determine the temperature that provides the optimal balance of optical and electrical properties for specific applications. In this study, we evaluated the subcell and top cell structures separately for monolithic c-Si HJT/perovskite tandem solar cells. The optimization of deposition temperature for the indium tin oxide (ITO) layer in the subcell structure was conducted using six different temperature values (room temperature (25 °C), 150 °C, 175 °C, 200 °C, 225 °C, and 250 °C). Subsequently, HJT solar cells were fabricated, and photovoltaic parameters were measured. Perovskite cells were then produced on the two HJT cells displaying the highest and lowest efficiency values to generate tandem solar cells.

## 2. Materials and Methods

### 2.1. ITO Film Deposition

ITO films deposited at different temperatures (RT, 150 °C, 175 °C, 200 °C, 225 °C, and 250 °C) on soda-lime glass (surface: 2.5 × 2.5 cm^2^, thickness: 1.1 mm) to investigate their structural, optical, and electrical properties. In the manufacturing process of the HJT solar cell, the temperature of the ITO layer is automatically regulated using temperature sensors and heaters. This automated control ensures precise and consistent temperature conditions, which are crucial for achieving the desired material properties and device performance. The films were deposited using direct current (DC) magnetron sputtering integrated into a physical vapor deposition (PVD) system (Meyer Burger Technology AG, Thun, Switzerland) operating at 13.56 MHz. A 99.999% (5N) purity ITO sputtering target (Kurt J. Lesker Company, Jefferson Hills, PA, USA) was employed for the film deposition process, resulting in ITO films with a thickness of 100 nm.

Prior to placing the glass substrates into the PVD chamber, they were cleaned in ultrasonic baths containing acetone for 5 min, ethanol for 5 min, and distilled water for 10 min, followed by drying with nitrogen (N_2_) gas. The cleaned substrates were then introduced into the PVD system, and the system was allowed to reach a working pressure of 7.3 × 10^−6^ mbar. A deposition pressure of 2.1 × 10^−2^ mbar was maintained by continuously supplying argon (Ar) and oxygen (O_2_) to the chamber. Oxygen O_2_ gas was introduced during the sputtering process to control the stoichiometry of the deposited ITO film. The presence of oxygen accelerated the oxidation process and created the desired crystal structure. By adjusting the ratio of oxygen to argon gases, the oxygen content in the ITO film can be finely tuned, which is critical for achieving the desired electrical and optical properties. ITO films were deposited via DC magnetron sputtering at different temperatures while keeping the gas ratios (Ar: 200 sccm and O_2_: 3.3 sccm) and power (1850 W) constant. To prevent contamination, the system was initially operated in a vacuum before loading the substrates, and the process was then carried out. Table 1 shows these deposition parameters and their corresponding notation.

### 2.2. Fabrication of Solar Cells

A. Silicon Heterojunction solar cell (Subcell)

To investigate the effects of ITO films deposited at different temperatures on HJT solar cells, random pyramid-structured n-type crystalline silicon (c-Si) wafers with a thickness of 180 µm and a sheet resistance of 3–5 Ω/sq were fabricated using the Czochralski (CZ) method. To create the HJT solar cell, the oxide layer on the surface of the c-Si wafer was removed using hydrofluoric (HF) solution (Merck KGaA, Darmstadt, Germany), followed by rinsing with deionized water and drying with nitrogen gas to complete the cleaning process of the c-Si wafer. Subsequently, a total of 20 nm of intrinsic hydrogenated amorphous silicon (i) a-Si: H/n-type (n) a-Si: H layers were deposited on the front side of the Si wafer, while 20 nm of (i) a-Si H /p-type (p) a-Si: H layers were deposited on the rear side using plasma-enhanced chemical vapor deposition (PECVD) (Meyer Burger Technology AG, Thun, Switzerland). For the (i) a-Si: H layer, silane (SiH_4_) and hydrogen (H_2_) gases were used, for the (n) a-Si H layer, phosphine (PH_3_), SiH_4_, and H_2_ gases were employed, and for the (p) a-Si: H layer, trimethyl-boron (TMB), SiH_4_, and H_2_ gases were utilized.

**Figure 1:**
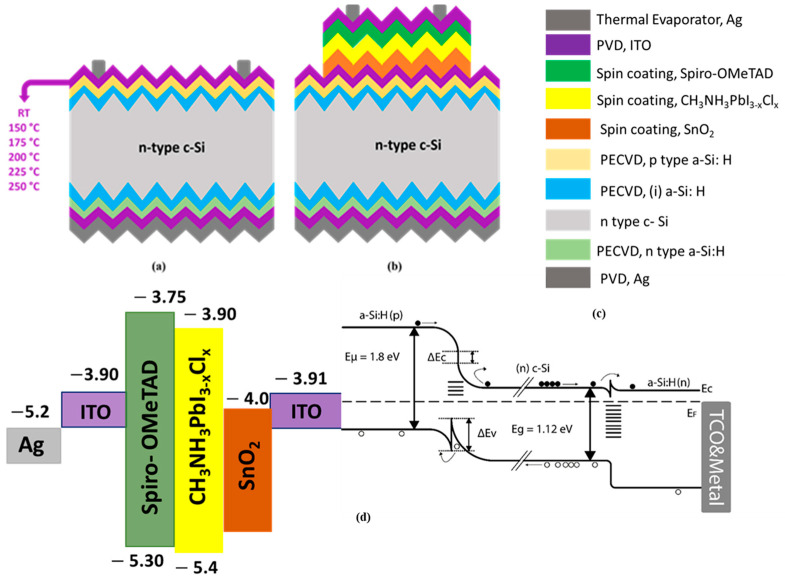
HJT solar cell (2.5 × 2.5 cm^2^) (**a**), HJT/perovskite tandem solar cell (active area: 1.45 cm^2^) (**b**), thin film colors and deposition methods (**c**), HJT/perovskite tandem solar cell eV diagram (**d**).

On the rear surface, deposition of the n-type a-Si: H layer was followed by the deposition of 40 nm ITO and 220 nm silver (Ag) layers using the PVD system. After coating the p-type a-Si: H layer, the front surface coatings were completed with the deposition of ITO films at different temperatures. Finally, finger and busbar patterns were printed on the front surface of the HJT solar cells using Ag paste via the screen print method (ASM Assembly Systems, Munich, Germany), and the solar cell was completed by firing it at 200 °C for 10 min [28,29]. Figure 1a provides a schematic representation and eV diagram [30] of the HJT solar cell.

B. Perovskite (Top cell) on silicon heterojunction solar cell

The solution-based steps for the production of the top cell began once the production of the subcells had been completed. All materials used in this study were purchased from commercial sources and used as received without any purification process. In order to maintain the purity of the materials, high-purity chemicals and solvents were selected. Furthermore, the inert (Ar) glove box system (Nanovak, Ankara, Turkey) in which these materials were produced both maintained the stability of the unstable materials against temperature and humidity and prevented possible contamination by keeping dust, particles, and contaminants under control. The ETL was synthesized using SnO_2_ collodial solution (Alfa Aesar, Haverhill, MA, USA) and isopropyl alcohol (IPA). For the perovskite layer, methylammonium iodide (MAI), lead chloride (PbCl_2_), lead iodide (PbI2), and anhydrous dimethylformamide (DMF) were used. HTL was formed with spiro-OMeTAD, tBP, Li-TFSI, 99.8% chlorobenzene, and 99.8% acetonitrile, following a specific sequence (all materials for depositing HTL and Perovskite layer were obtained from Sigma Aldrich (St. Louis, MO, USA). Instead of encapsulation, ITO (99.999%) was deposited on HTL to protect the cell. Finally, Ag (99.99%) was used as a metallization material. Top cell production was mainly solution based with spin coating. To facilitate this process, each material was presynthesized and filtered before cell production.

The SnO_2_ colloid solution was dissolved in 10 µL of isopropanol with magnetic stirring for 2 h at room temperature. Subsequently, filtration was performed to remove particulate matter and impurities from the solution using a 0.45 μm (PTFE) filter. The solution prepared on the spin coating was taken with the help of a pipette and then coating was performed on ITO by rotating it at 3000 rpm for 30 s. After that, the coating was annealed at 150 °C for 30 min. The material was then transferred to the glove box to apply a perovskite layer coating. Before coating the perovskite on the coated ETL layer, it was heated at a low temperature on the hot plate for a while. The ETL layer was coated with a formulation of CH_3_NH_3_PbI_3−x_Cl_x_ in a molar ratio of 1:1:4 containing methylammonium iodide (MAI), PbCl_2_ and PbI_2_ in DMF. The coated layer was then heated on a hot plate at a low temperature of 60 °C for one hour. Next, perovskite solution was dripped onto the ETL layer through a 0.45 μm (PTFE) filter and spun at 4000 rpm for 45 s. Then, a 70 °C annealing process was performed on a hot plate. Once the perovskite layer was annealed, it was allowed to cool down before the application of the prepared Spiro-OMeTAD solution. Using the dynamic coating method, the solution was coated at a speed of 4000 rpm for 30 s. Upon the completion of the monolithic tandem cell, no encapsulation procedure was implemented.

The use of an ITO layer instead of encapsulation in a tandem solar cell reflected the trade-off between protection, electrical performance, transparency, and cost-effectiveness. This particular design choice responded to specific application requirements and the desired balance between these factors. The final stage of the cell, the front contacts, was carried out using a mask with a thickness of approximately 150 nm thickness under a pressure of 10^−6^ Torr. The completed monolithic tandem structure is shown in Figure 1b.

C. Characterization

The structural, optical, and electrical parameters of the ITO films used in HJT solar cells were subjected to a detailed analysis using the films deposited. The structural characteristics of the ITO films and the layers of the perovskite solar cells used as top cells were investigated via X-ray diffraction (Pan analytical-XRD, Malvern, UK, J.J CuKα radiation, λ = 0.15406 nm). The measurement of the thickness and optical properties of the ITO films was conducted using a Woollam V-Vase ellipsometer (Lincoln, NE, USA). The thickness measurement of the layers in perovskite solar cells was carried out using a Bruker Dektak XT profilometer (Billerica, MA, USA) device.

The electrical properties of the ITO films were determined by employing a contactless sheet resistance measurement system, EddyCus^®^ TF lab 4040 Hybrid (Dresden, Germany). Furthermore, cross-sectional images of the c-Si HJT/perovskite tandem solar cells were examined using field emission-scanning electron microscopy, FE-SEM (HITACHI SU5000, Tokyo, Japan).

## 3. Results

### 3.1. Effect of Temperature on the Structural Properties of ITO Films

**Figure 2:**
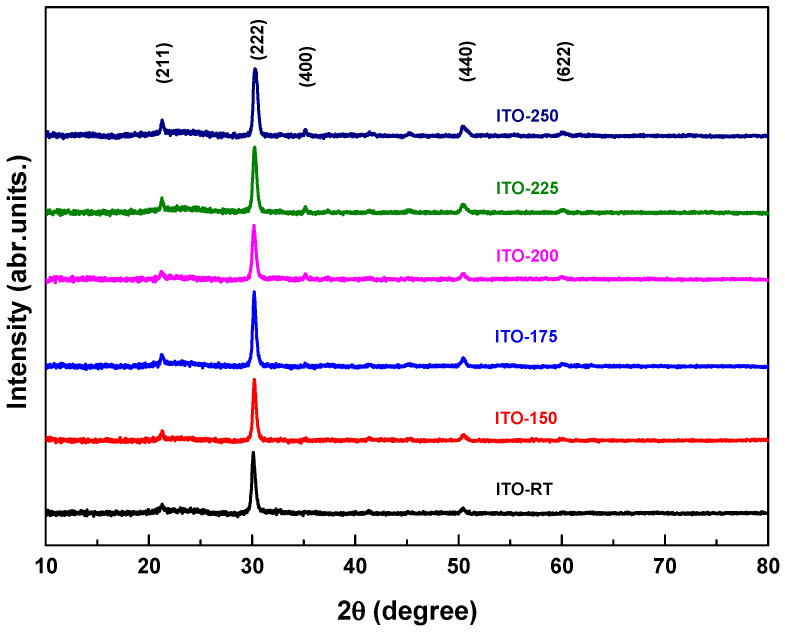
XRD patterns of ITO-RT, ITO-150, ITO-175, ITO-200, and ITO-250 films samples.

Figure 2 shows the XRD analysis of ITO films deposited at different temperatures (RT, 150, 175, 200, 225, 250 °C). ITO films were scanned by selecting the 2θ scanning range of 10 to 80°. According to the analysis, the diffraction peaks (211), (222), (400), (440), and (622) corresponded to cubic ITO [31]. According to the analysis, the diffraction peaks (211), (222), (400), (440), and (622) corresponded to cubic ITO and all films showed polycrystalline properties [31,32,33,34]. While the (400) peak was not visible in the ITO-RT, ITO-150, and ITO-175 samples, it was clearly seen when the temperature was applied above 175 °C (Figure 2). This meant that the applied temperature treatment improved the arrangement of atoms, which lead to a better crystal structure [35]. Table 2 shows the structural parameters of ITO films deposited at different temperatures. The structural parameters of ITO films were calculated according to the diffraction peak (222) plane of the highest intensity. It should be noted that the orientation of ITO (222) and (400) diffraction peaks can be changed by increasing the film ratio [36] and temperature [37]. According to (222) plane, the ITO-150 sample had the lowest full width half max (FWHM) value (7.18 × 10^−3^ rad), while the FWHM value of the ITO-250 was the highest (8.67 × 10^−3^ rad). In addition, a continuous increase was observed in the FWHM values of ITO films deposited above 150 °C as the temperature increased. The 2θ values of the all ITO-RT, ITO-150, ITO-175, ITO-200, and ITO-250 samples in the (222) orientation were found to be 30.14°, 30.23°, 30.22°, 30.19°, 30.25°, and 30.32°, respectively ((ICSD Card No: 98-005-0848 (ITO-RT, ITO-150, and ITO-200), ICSD Card No: 98-005-0847 (ITO-175), and ICSD Card No: 98-005-0849 (ITO-225, and ITO-250)).

**Figure 3:**
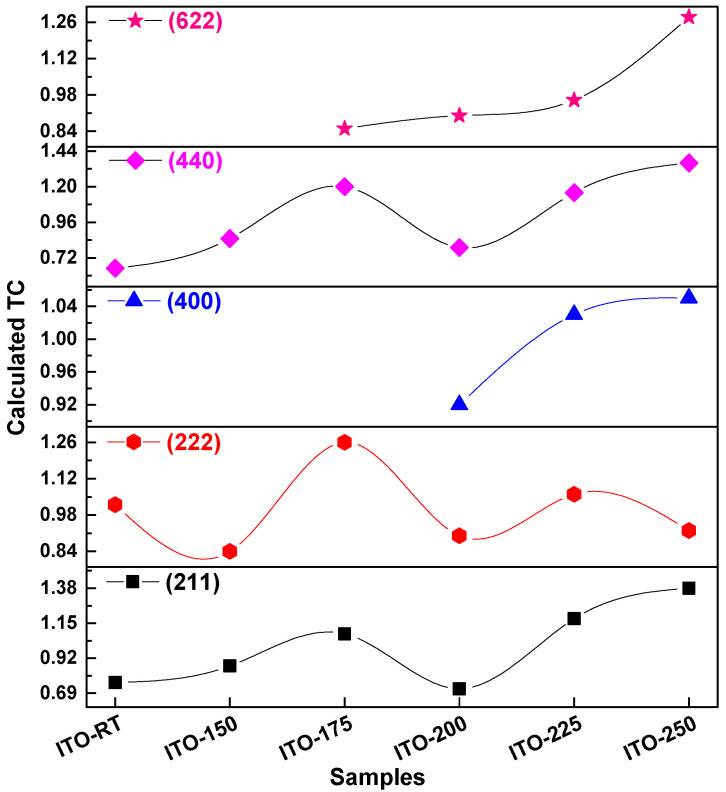
Texture coefficients (TC) of ITO films produced at different temperatures calculated from XRD patterns.

The crystal size was calculated using Equation (1) where k is the shape factor (0.9), λ is the X-ray wavelength, *β* is the broadening of the diffraction line peak at an angle of 2θ at FWHM in radians measured using Gaussian distribution, and θ is the Bragg angle. Table 2 shows the crystal size and *β* values of ITO films deposited at different temperatures. It was observed that the crystal size and *β* values were inversely proportional, and the β decreased clearly while the crystal size increased (Figure 3). In addition, as shown in Table 2, while a decrease in crystal size was observed with increasing temperature from 150 to 250 °C, the best crystal size was determined as 20 nm at 150 °C [38]. Although this shows that ITO samples crystallize better at 150 °C, different characterization methods are needed since the values were very close to each other.

(1) $$D=\frac{k\lambda }{\beta \cos\theta }\;$$

The dislocation density (*δ*), microstrain (*ε*) and number of crystallites per unit area (N) were calculated using Equations (2)–(4) and are shown in Table 2 [39]. The t value in Equation (4) is the film thickness. At 150 °C and above, a steady increase in the dislocation density of the ITO films deposited was observed. The dislocation densities of ITO-RT and ITO-200 were equal and calculated as 3.1 × 10^−3^ lines/nm^2^. While the lowest dislocation density was calculated for ITO-150 (2.5 × 10^−3^ lines/nm^2^), the highest dislocation density was calculated for the ITO-250 (3.9 × 10^−3^ lines/nm^2^). The microstrain parameters showed a steady increase in the ITO films deposited at 150 °C and above. The microstrain parameter of the ITO-RT and ITO-200 was equal and calculated as 1.9 × 10^−3^. While the lowest microstrain parameter was calculated in the ITO-150 (1.7 × 10^−3^), the highest microstrain was calculated in the ITO-250. The number of crystallites per unit area showed a steady increase in ITO films deposited at 150 °C and above. The number of crystallites per unit area of ITO-RT and ITO-200 was equal and calculated as 1.7 × 10^−2^ nm^−2^. In addition, the lowest number of crystallites per unit area was calculated in the ITO-150 (1.3 × 10^−2^ nm^−2^), and the highest number of crystallites per unit area was calculated in the ITO-250 (2.4 × 10^−2^ nm^−2^).

(2) $$\delta =\frac{1}{D^{2}}\;$$

(3) $$\varepsilon =\frac{\beta \cos\theta }{4}$$

(4) $$N=\frac{t}{D^{3}}\;$$

The temperature coefficients (TC) for diffraction peak (211), (222), (400), (440), and (622) were calculated using ICSD cards and Equation (5) [40].

(5) $$TC_{hkl}=\frac{\frac{I_{means}\;\left(hkl\right)}{I_{0}\left(hkl\right)}}{\frac{1}{N}\sum_{\overset{´}{h}\overset{´}{k}\overset{´}{l}}\frac{I_{meas}\left(\overset{´}{h}\overset{´}{k}\overset{´}{l}\right)}{I_{0}\left(\overset{´}{h}\overset{´}{k}\overset{´}{l}\right)}}$$

where *I_means_*, *I*_0_ and *N* are defined as the measured intensities of each *(hkl)* point, the theoretical relative intensity given in the ICSD cards and the number of reflections considered, respectively. For diffraction peaks (211), (222), and (440), we used N = 6, for (622) we used N = 4, and for (400) we used N = 3. The N values corresponding to the XRD peaks seen in Figure 2 were used for each ITO thin film. Figure 3 illustrates the texture coefficients of ITO films deposited at different temperatures, with corresponding values given in Table 3. In addition, Table 3 shows the *I_means_* corresponding to the diffraction peaks (211), (222), (400), (440), and (622) and the *I*_0_ values obtained from the ICSD card. Considering the TC*_hkl_* values, it was observed that the orientations of the ITO films were towards the diffraction peaks (211), (222), and (440). Additionally, it was noted that there was a consistent increase in TC*_hkl_* values for TC*_400_* and TC*_622_* as the temperature rose.

### 3.2. Effect of Temperature on the Optical Properties of ITO Films

**Figure 4:**
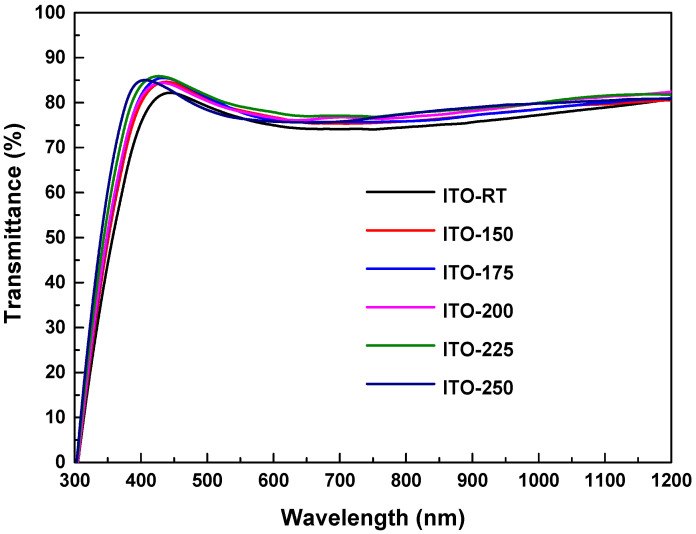
Optical transmittance spectra versus wavelength for ITO deposited on glass substrates via the PVD system at ITO-RT, ITO-150, ITO-175, ITO-200, and ITO-250 different temperatures.

**Figure 5:**
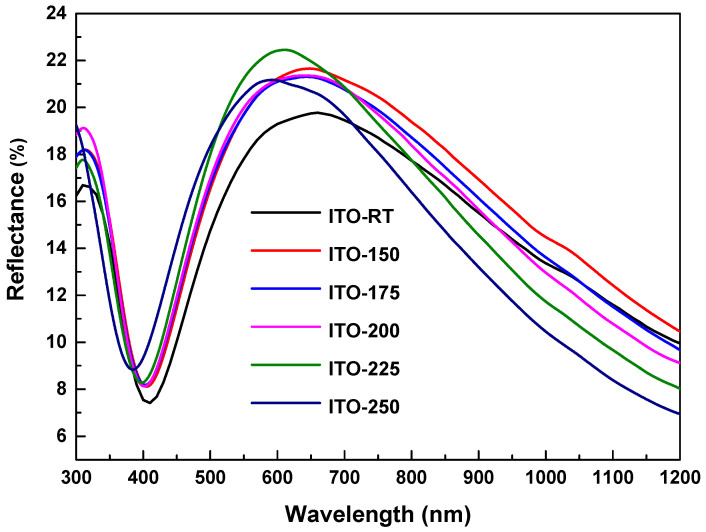
Optical reflectance spectra of ITO films deposited on glass substrates via the PVD system at ITO-RT, ITO-150, ITO-175, ITO-200, and ITO-250 temperatures, plotted against wavelength.

The optical transmittance and reflectance spectra versus wavelength of ITO deposited by the PVD system on glass substrates at different temperatures are shown in Figure 4 and Figure 5. The results in Table 4 include the optical properties of the film with glass substrate. The transmittance spectra of the films deposited at different temperatures showed that they were highly transparent in the visible region of the electromagnetic spectrum. In the wavelength range of 400 to 1200 nm, ITO deposited at different temperatures showed an average transmittance of over 77% and an average reflectance of over 14% (Table 4). It was also observed that while the average transmittance value increased with increasing temperatures, it decreased at 225 °C. The highest average transmittance was measured in ITO-200 (80%), and the lowest transmittance was measured in ITO-RT (77%). The increase in temperature increased the transmittance in the near-infrared region, while it decreased the transmittance at higher wavelengths. The increase in reflectance in the long wavelength region may be attributed to an increase in carrier concentration, likely caused by oxygen deficiency at high temperatures [41].

**Figure 6:**
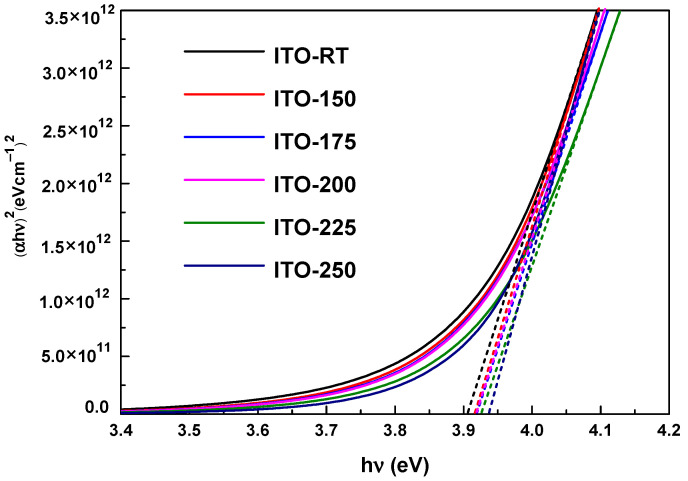
Band gap measurement of ITO deposited at different temperatures, obtained by plotting *hν* versus (αhν)^2^. The optical band gap of the deposited ITO films was calculated to be between 3.90 eV and 3.93 eV (The band gap was calculated using fitted curves, which are represented by dashed lines in the figure).

The optical absorption coefficient in the visible region (α) and the energy of the incident photon (hν) are needed to determine the optical band gap (Eg) in semiconductor materials [42]. In this study, the band gap measurement of ITO deposited at different temperatures was obtained by plotting *hν* versus (*αhν*)^2^ (Figure 6). The optical bandgap obtained from the deposited ITO films was calculated to be between 3.90 eV and 3.93 eV. Energy band gap values are given in Table 4. According to the results obtained, an increase in the bandgap of ITO films was observed as the temperature increased. The increase in the bandgap of ITO can be attributed to the shift of the absorption edge towards the near UV region. This phenomenon, commonly referred to as the Burstein–Möss (B-M) effect [43], may arise from factors such as higher deposition temperatures and increased carrier concentrations.

### 3.3. Effect of Temperature on the Electrical Properties of ITO Films

**Figure 7:**
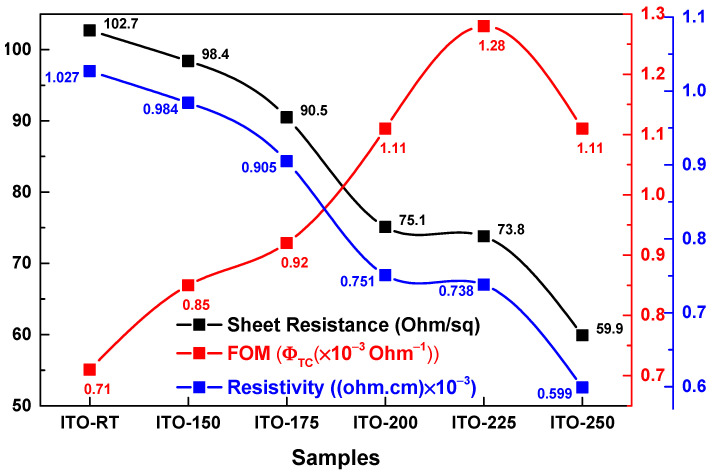
Sheet resistance, FOM, and resistivity graph of ITO films as a function of the temperature. The FOM values calculated from Haacke’s method [49], which optimally characterized the optical and electrical properties of ITO films through electrical sheet resistance and optical transmittance.

Transmittance values at 550 nm were used to calculate the temperature-dependent Figure of Merit (FOM) of the ITO films (Table 4). Additionally, the sheet resistance, FOM, and resistivity of the ITO films were plotted [44]. According to Haacke, the optical and electrical properties of transparent conductive oxide (TCO) films are best characterized via electrical sheet resistance and optical transmittance. Therefore, the FOM value was defined as an important parameter for evaluating the performance and determining the material quality of the films [45,46,47,48]. It is calculated using Equation (6) below.

(6) $$\Phi_{TC}=T^{10}/R_{sh}\;$$

where T is the transmittance and $R_{sh}$ is the sheet resistance of ITO films [49]. Figure 7 shows the $\Phi_{TC}$ and $R_{sh}$ values of ITO deposited at different temperatures. It was seen that $\Phi_{TC}$ values increased as the deposition temperature increased (except 250 °C). ITO-225 was calculated to have the best FOM value (1.28 × 10^−3^ Ohm^−1^). Figure 7 shows the $R_{sh}$ values of ITOs deposited at different temperatures. The electrical properties of ITO thin films depended on the deposition parameters such as film composition, temperature, and oxygen content. It was observed that a steady decrease in $R_{sh}$ and resistivity values occurred with the increase in the temperature. Among the ITO films deposited, the highest $R_{sh}$ value was 102.7 Ohm/sq in ITO-RT, and the lowest $R_{sh}$ value was 59.9 Ohm/sq in ITO-250. In the resistivity values, the highest value was obtained as 102.7 × 10^−3^ ohm.cm in ITO-RT film, and the lowest value was obtained as 59.9 × 10^−3^ ohm.cm in ITO-250. The electrical properties of the ITO films were improved as the temperature increased.

### 3.4. Effect of Temperature on the HJT Solar Cell Parameters

The optimization of the deposition temperature proved to be a crucial factor for the optical and electrical properties of ITO thin films, and also for the cell parameters of HJT solar cells. Table 5 summarizes the key parameters obtained at each ITO deposition temperature, showcasing notable variations in the solar cell performance. Notably, at room temperature, the efficiency remained suboptimal, indicating clearly insufficient V_OC_. It is well known that V_OC_ is strongly correlated with conductivity and is inversely proportional to R_SH_ values (Figure 7) of the TCO layer. Based on that, the lowest V_OC_ value of the ITO-RT sample can be easily explained.

**Figure 8:**
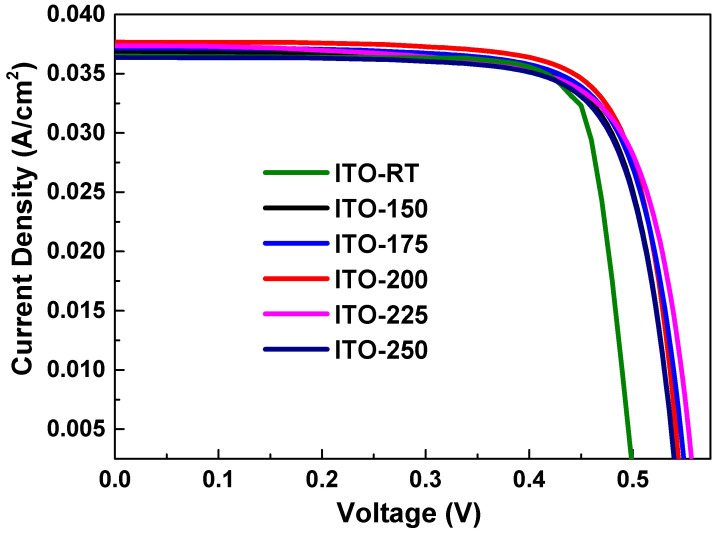
Current density (J)-voltage (V) graphs of HJT solar cells with ITO layers deposited at different temperatures: ITO-RT, ITO-150, ITO-175, ITO-200, and ITO-250.

To improve the monolithic tandem cell efficiency of the HJT cells shown in Figure 8, the top perovskite cell production stage was initiated taking into account the Jsc and FF values in Table 5 and the subcells with the lowest and highest efficiency values as reference.

### 3.5. Structural and Morphological Properties of Monolithic c-Si HJT/perovskite Tandem Solar Cell

**Figure 9:**
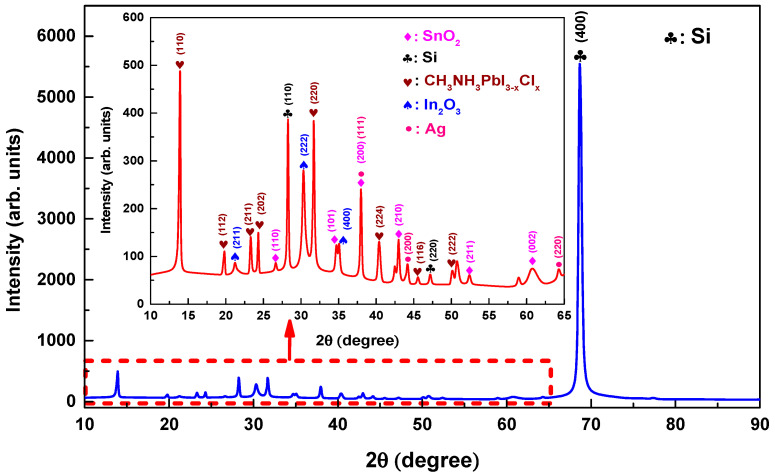
The XRD results for the monolithic c-Si heterojunction perovskite tandem solar cell, including a focus on the top cell.

Structural analyses of the films of the tandem cell were carried out using XRD with the CuKα (λ = 0.15406 nm) X-ray source. The scanning range was 2θ = 10–90°. The results of the XRD analysis for the tandem films are presented in Figure 9. Measurements were taken in ambient air. As the Si peak at 2θ 68.66 was more dominant than the intensity of the other peaks, the red area has been zoomed in and is shown in the figure. The following Si peaks were observed: 2θ = 28.26° (110), 47.18° (220), and 68.66° (400). The Ag peaks were at 37.96° (111), 44.16° (200), and 64.26° (220). SnO_2_ peaks were located at 26.64° (110), 34.17° (101), 37.96° (200), 42.96° (210), 52.38° (211), and 60.72° (002) [50]. The In_2_O_3_ peaks were at 21.23° (211), 30.3° (222), 35.1° (400), and 21.23° [51,52]. Finally, CH_3_NH_3_PbI_3_-_x_Cl_x_ peaks were found at 13.92° (110), 19.82° (112), 23.32° (211), 24.32° (202), 31.68° (220), 40.38° (224), 45.54° (116), and 50.14° (222) [53,54]. No X-ray diffraction peaks were observed from the spiro-OMeTAD HTL, indicating that the material was amorphous [55].

**Figure 10:**
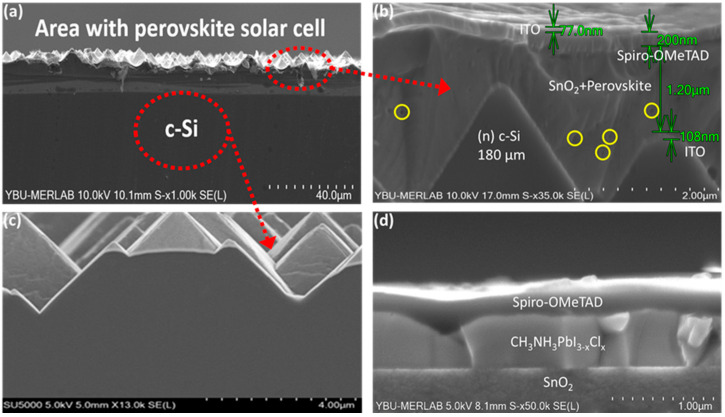
c-Si HJT/perovskite tandem solar cell SEM images. (**a**) Random pyramid-structured n-type c-Si and area with perovskite solar cell, (**b**) cross-section view of tandem layers and thickness (the yellow circles), (**c**) the cross-section of HJT solar cell, (**d**) top perovskite cell structure.

The SEM measurements in the context of perovskite/c-Si HJT monolithic tandem solar cells has provided information on the morphology, structure, and quality of individual layers and interfaces within the solar cell structure. Figure 10a illustrates the random pyramid structure of n-type c-Si and perovskite solar cells. While Figure 10c exhibits the cross-section of a HJT cell, Figure 10d shows the top perovskite cell structure. SEM images of the perovskite top cell exhibited structural defects and pinholes in areas where the textured HJT sublayer did not entirely fill the valleys; these regions of pinholes and defects had a negative impact on the efficiency of the monolithic tandem solar cell. The silicon heterojunction solar cell (subcell) comprised a 180 µm thick n-type crystalline silicon (c-Si) layer, 10 nm thick amorphous silicon (a-Si: H) thin films, and 100 nm thick indium tin oxide (ITO) layers. The layer thicknesses of the top cell were as follows: ITO 77 nm, Spiro-OMeTAD 200 nm, and SnO_2_+perovskite 1200 nm, as illustrated in Figure 10b.

### 3.6. c-Si HJT and Monolithic c-Si HJT/Perovskite Tandem Solar Cell Efficiency

**Figure 11:**
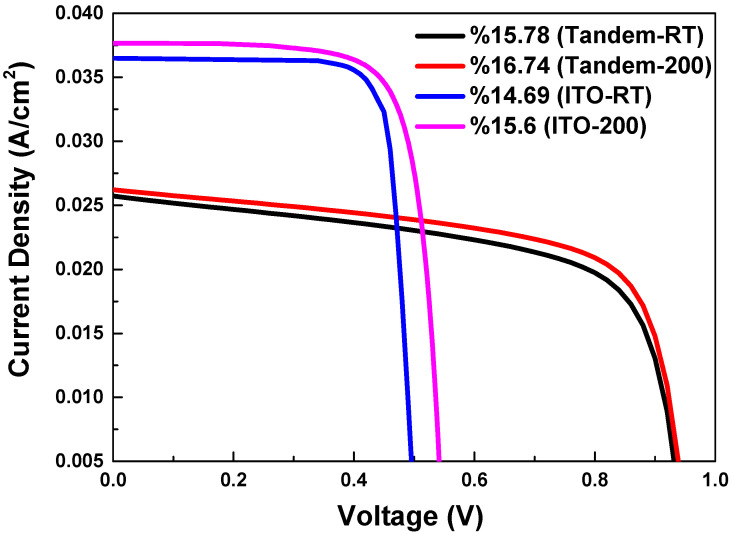
A comparison of the efficiency of a monolithic tandem solar cell comprising n-type c-Si/ITO/SnO_2_/CH_3_NH_3_PbI_3_−xClx/Spiro-OMeTAD/ITO/Ag and a c-Si HJT solar cell is presented. The tandem solar cell’s conversion efficiencies utilizing ITO deposited at room temperature and 200 °C are designated as Tandem-RT (15.78%) and Tandem-200 (16.74%), respectively.

Figure 11 shows the efficiency of a monolithic tandem solar cell composed of n-type c-Si/ITO/SnO_2_/CH_3_NH_3_PbI_3−x_Cl_x_/Spiro-OMeTAD/ITO/Ag and a c-Si HJT solar cell. The conversion efficiency of the tandem solar cell, utilizing ITOs deposited at room temperature and 200 °C, was measured as 15.78% and 16.74%, respectively. The results indicated that the crystallinity of materials used in tandem solar cells was significantly affected by temperature optimization during deposition processes. Higher temperatures generally led to better crystallinity, which typically resulted in lower defect densities. This, in turn, can facilitate more efficient charge carrier transport and collection, ultimately contributing to higher overall efficiency.

## 4. Conclusions

In conclusion, this study investigated the effects of temperature optimization on the ITO film in the subcell of a monolithic HJT/perovskite tandem solar cell architecture. Through optimization, the optimal temperature for the ITO film within the subcell was determined to be 200 °C. The efficiency of the Tandem-200 configuration was found to be 16.74%, which represented a significant improvement over the efficiency of the Tandem-RT configuration, which stood at 15.78%. The optimization of the temperature in the subcell resulted in an increase in efficiency of approximately 1%. The light transmittance exceeded 80% and the sheet resistance was 75.1 Ω/sq for ITO deposited at 200 °C. Analysis revealed the presence of structural defects and pinholes in the perovskite top cell, as well as areas on the textured surface of the HJT bottom cell where the perovskite did not uniformly cover. Additionally, while thicker layers of the perovskite top cell initially enhanced light absorption, they also introduced various limiting factors, including increased self-absorption, losses in charge transport, optical losses, and stability issues. The presence of pinholes, defects, and thick cell structures collectively impaired the efficiency of monolithic tandem solar cells. In general, the response of ITO to temperature changes has a direct impact on the overall performance and durability of tandem solar cells. Optimizing the electrical and optical properties of ITO, as well as its thermal management capability, enabled cells to operate at high efficiency. Minimizing temperature stress and expansion mismatches maintained the structural integrity of the cells and ensured their long lifetime. Therefore, optimizing ITO against temperature effects is critical to improving the overall performance of tandem solar cells. Recent progress in silicon HJT/perovskite tandem solar cells has centered on optimizing the layers in both the top and bottom cells and incorporating innovative materials and diversifying the types of silicon cells used as the bottom layer. Future research can aim to develop commercially viable methods to improve the interface between HJT and perovskite solar cells, such as improving the TCO structures used as buffer layers and developing techniques to enable the homogeneous deposition of perovskite solar cells onto HJT solar cells.

## Figures and Tables

**Table 1:** Deposition parameters and corresponding sample notations of ITO films.

Sample Notation	Ar Flow (sccm)	O_2_ Flow (sccm)	Deposition Power (W)	Deposition Temperature (°C)
ITO-RT	200	3.3	1850	RT
ITO-150	200	3.3	1850	150
ITO-175	200	3.3	1850	175
ITO-200	200	3.3	1850	200
ITO-225	200	3.3	1850	225
ITO-250	200	3.3	1850	250

**Table 2:** FWHM (*β*), Bragg angle (θ), crystal size (D), dislocation density (*δ*), microstrain (*ε*), and number of crystallites per unit area (*N*) values of ITO films.

Sample Name	*β* (Rad)	*θ* (Degrees)	cos*θ*	*D* (nm)	*δ* (Lines/nm^2^) (×10^−3^)	*ε* (×10^−3^)	*N* (nm^−2^) (×10^−2^)
ITO-RT	7.90 × 10^−3^	15.0699	0.9656	18	3.1	1.9	1.7
ITO-150	7.20 × 10^−3^	15.1153	0.9654	20	2.5	1.7	1.3
ITO-175	7.30 × 10^−3^	15.1082	0.9654	19	2.8	1.8	1.5
ITO-200	7.90 × 10^−3^	15.0948	0.9655	18	3.1	1.9	1.7
ITO-225	8.20 × 10^−3^	15.1266	0.9654	17	3.5	2	2
ITO-250	8.70 × 10^−3^	15.1621	0.9652	16	3.9	3.1	2.4

**Table 3:** Texture coefficient values calculated for the 5 main peaks obtained from XRD.

Sample	Diffraction Peaks					
	(211)	(222)	(400)	(440)	(622)	
ITO-RT	10.3	100	30.5	37.3	27.4	I_0_
ITO-150	10.3	100	30.5	37.3	27.4	
ITO-175	10.7	100	30.6	37.1	27.4	
ITO-200	10.3	100	30.5	37.3	27.4	
ITO-225	11.1	100	30.5	36.8	26.9	
ITO-250	11.1	100	30.5	36.8	26.9	
ITO-RT	319.6	2373.8	-	172.7	-	Imeans
ITO-150	364.8	1944.7	-	225.4	-	
ITO-175	470.4	2919.7	-	318	100	
ITO-200	302.8	2098.4	190.8	211	105.1	
ITO-225	533.3	2469.8	214.3	303.7	110.8	
ITO-250	623.3	2128.4	217.2	356	147.3	
ITO-RT	0.76	1.02	-	0.65	-	Texture Coefficient (TC)
ITO-150	0.87	0.84	-	0.85	-	
ITO-175	1.08	1.26	-	1.20	0.85	
ITO-200	0.72	0.90	0.92	0.79	0.90	
ITO-225	1.18	1.06	1.03	1.16	0.96	
ITO-250	1.38	0.92	1.05	1.36	1.28	

**Table 4:** Transmittance, reflectance, and energy band gaps of ITO films.

Sample Name	Average Transmittance (%) (400–1200 nm)	Average Reflectance (%) (400–1200 nm)	Eg (eV)	Transmittance (%) (550 nm)
ITO-RT	77	15	3.9	77
ITO-150	78	16	3.91	78
ITO-175	78	16	3.91	78
ITO-200	80	15	3.91	78
ITO-225	79	15	3.92	79
ITO-250	79	14	3.93	76

**Table 5:** Cell parameters of temperature-dependent ITO layers deposited on identical HJT solar cells.

Sample Name	Efficiency (%)	Current Density, J_SC_ (A/cm^2^)	Open Circuit Voltage, V_OC_ (V)	Fill Factor, FF (%)
ITO-RT	14.69	0.0365	0.5008	80.39
ITO-150	15.05	0.0368	0.5430	75.40
ITO-175	15.30	0.0372	0.5520	74.40
ITO-200	15.60	0.0377	0.5460	75.70
ITO-225	15.20	0.0374	0.5590	72.50
ITO-250	14.90	0.0364	0.5430	75.40

## Data Availability

Data are contained within the article.

## References

[1] Fraunhofer Institute for Solar Energy Systems. ISE. www.cell-to-module.com.

[2] Hafshejani M.T., Keshavarzi R., Mirkhani V., Moghadam M., Tangestaninejad S., Mohammadpoor-Baltork I. (2024). Waste Carbon-Based Toner Protection Layer on CsPbBr3 Perovskite Photoanodes for Efficient and Stable Photoelectrochemical Water Oxidation. Int. J. Hydrogen Energy.

[3] NREL Best Research-Cell Efficiency Chart. www.nrel.gov/pv/cell-efficiency.html.

[4] Richter A., Hermle M., Glunz S.W. (2013). Reassessment of the Limiting Efficiency for Crystalline Silicon Solar Cells. IEEE J. Photovolt..

[5] Jacak J.E., Jacak W.A. (2022). Routes for Metallization of Perovskite Solar Cells. Materials.

[6] Laska M., Krzemińska Z., Kluczyk-Korch K., Schaadt D., Popko E., Jacak W.A., Jacak J.E. (2020). Metallization of Solar Cells, Exciton Channel of Plasmon Photovoltaic Effect in Perovskite Cells. Nano Energy.

[7] Jošt M., Kegelmann L., Korte L., Albrecht S. (2020). Monolithic Perovskite Tandem Solar Cells: A Review of the Present Status and Advanced Characterization Methods toward 30% Efficiency. Adv. Energy Mater..

[8] Sahli F., Werner J., Kamino B.A., Bräuninger M., Monnard R., Paviet-Salomon B., Barraud L., Ding L., Diaz Leon J.J., Sacchetto D. (2018). Fully Textured Monolithic Perovskite/Silicon Tandem Solar Cells with 25.2% Power Conversion Efficiency. Nat. Mater..

[9] Wu Y., Zheng P., Peng J., Xu M., Chen Y., Surve S., Lu T., Bui A.D., Li N., Liang W. (2022). 27.6% Perovskite/C-Si Tandem Solar Cells Using Industrial Fabricated TOPCon Device. Adv. Energy Mater..

[10] Bailie C.D., McGehee M.D. (2015). High-Efficiency Tandem Perovskite Solar Cells. Mrs Bull..

[11] Gerritsen S., Nguyen H.V., Creatore A. Studying Degradation of Perovskite Solar Cells in Ambient Atmosphere Using Photoluminescence Spectroscopy 2019. https://api.semanticscholar.org/CorpusID:202609581.

[12] Li B., Li Y., Zheng C., Gao D., Huang W. (2016). Advancements in the Stability of Perovskite Solar Cells: Degradation Mechanisms and Improvement Approaches. RSC Adv..

[13] Zhao J., Wang A., Altermatt P.P., Green M.A., Rakotoniaina J.P., Breitenstein O. High Efficiency PERT Cells on N-Type Silicon Substrates. Proceedings of the Conference Record of the Twenty-Ninth IEEE Photovoltaic Specialists Conference.

[14] Müller J., Rech B., Springer J., Vanecek M. (2004). TCO and Light Trapping in Silicon Thin Film Solar Cells. Sol. Energy.

[15] Altuntepe A., Olgar M.A., Erkan S., Hasret O., Keçeci A.E., Kökbudak G., Tomakin M., Seyhan A., Turan R., Zan R. (2021). Hybrid Transparent Conductive Electrode Structure for Solar Cell Application. Renew. Energy.

[16] Zan R., Olgar M.A., Altuntepe A., Seyhan A., Turan R. (2022). Integration of Graphene with GZO as TCO Layer and Its Impact on Solar Cell Performance. Renew. Energy.

[17] Chavan G.T., Kim Y., Khokhar M.Q., Hussain S.Q., Cho E.-C., Yi J., Ahmad Z., Rosaiah P., Jeon C.-W. (2023). A Brief Review of Transparent Conducting Oxides (TCO): The Influence of Different Deposition Techniques on the Efficiency of Solar Cells. Nanomaterials.

[18] Al-Kuhaili M.F. (2023). Transparent-Conductive and Infrared-Shielding WO_3_/Ag/WO_3_ Multilayer Heterostructures. Sol. Energy.

[19] Suwanboon S., Amornpitoksuk P., Haidoux A., Tedenac J.-C. (2008). Structural and Optical Properties of Undoped and Aluminium Doped Zinc Oxide Nanoparticles via Precipitation Method at Low Temperature. J. Alloys Compd..

[20] Zhang H., Qu F., Li H. (2022). Front Transparent Passivation of CIGS-Based Solar Cells via AZO. Molecules.

[21] Hussain S.Q., Kim S., Ahn S., Balaji N., Lee Y., Lee J.H., Yi J. (2014). Influence of High Work Function ITO: Zr Films for the Barrier Height Modification in a-Si: H/c-Si Heterojunction Solar Cells. Sol. Energy Mater. Sol. Cells.

[22] Han C., Zhao Y., Mazzarella L., Santbergen R., Montes A., Procel P., Yang G., Zhang X., Zeman M., Isabella O. (2021). Room-Temperature Sputtered Tungsten-Doped Indium Oxide for Improved Current in Silicon Heterojunction Solar Cells. Sol. Energy Mater. Sol. Cells.

[23] Kobayashi E., Watabe Y., Yamamoto T., Yamada Y. (2016). Cerium Oxide and Hydrogen Co-Doped Indium Oxide Films for High-Efficiency Silicon Heterojunction Solar Cells. Sol. Energy Mater. Sol. Cells.

[24] Yao Z., Duan W., Steuter P., Hüpkes J., Lambertz A., Bittkau K., Pomaska M., Qiu D., Qiu K., Wu Z. (2021). Influence of Oxygen on Sputtered Titanium-Doped Indium Oxide Thin Films and Their Application in Silicon Heterojunction Solar Cells. Sol. RRL.

[25] Messmer C., Tutsch L., Pingel S., Erath D., Schön J., Fell A., Goldschmidt J.C., Goraya B.S., Clement F., Lorenz A. (2022). Optimized Front TCO and Metal Grid Electrode for Module-integrated Perovskite–Silicon Tandem Solar Cells. Prog. Photovolt. Res. Appl..

[26] Ba L., Wang T., Wang J., Shen W. (2019). Perovskite/c-Si Monolithic Tandem Solar Cells under Real Solar Spectra: Improving Energy Yield by Oblique Incident Optimization. J. Phys. Chem. C.

[27] Sahoo N.G. (2021). New Generation Transparent Conducting Electrode Materials for Solar Cell Technologies. Mater. Res. Found..

[28] Seyhan A., Kartal E. (2023). Optical, Electrical and Structural Properties of ITO/IZO and IZO/ITO Multilayer Transparent Conductive Oxide Films Deposited via Radiofrequency Magnetron Sputtering. Coatings.

[29] Kartal E., Duran İ., Damgaci E., Seyhan A. (2023). Investigation of Structural, Optical, and Electrical Properties of Ito Films Deposited at Different Plasma Powers: Enhanced Performance and Efficiency in SHJ Solar Cells. Eurasian J. Sci. Eng. Technol..

[30] Zan R., Altuntepe A., Altan T., Seyhan A. (2021). Crystalline-Silicon Heterojunction Solar Cells with Graphene Incorporation. Sustainable Material Solutions for Solar Energy Technologies.

[31] Raoufi D., Taherniya A. (2015). The Effect of Substrate Temperature on the Microstructural, Electrical and Optical Properties of Sn-Doped Indium Oxide Thin Films. Eur. Phys. J. Appl. Phys..

[32] Lien S.-Y. (2010). Characterization and Optimization of ITO Thin Films for Application in Heterojunction Silicon Solar Cells. Thin Solid Films.

[33] Ogi T., Hidayat D., Iskandar F., Purwanto A., Okuyama K. (2009). Direct Synthesis of Highly Crystalline Transparent Conducting Oxide Nanoparticles by Low Pressure Spray Pyrolysis. Adv. Powder Technol..

[34] Zhang K., Zhu F., Huan C.H.A., Wee A.T.S. (1999). Effect of Hydrogen Partial Pressure on Optoelectronic Properties of Indium Tin Oxide Thin Films Deposited by Radio Frequency Magnetron Sputtering Method. J. Appl. Phys..

[35] Mustapha N., Alakhras A., Idriss H. (2020). Influence of Post-Deposition Annealing on the Indium Tin Oxide Thin Films Grown by Pulsed Laser Deposition. Dig. J. Nanomater. Biostruct..

[36] Thilakan P., Kumar J. (1997). Studies on the Preferred Orientation Changes and Its Influenced Properties on ITO Thin Films. Vacuum.

[37] Meng L., Dos Santos M.P. (1998). Properties of Indium Tin Oxide Films Prepared by Rf Reactive Magnetron Sputtering at Different Substrate Temperature. Thin Solid Films.

[38] Ezzeldien M., Gami F., Alrowaili Z.A., Shaaban E.R., El-Hagary M. (2022). The Influential Role of ITO Heat Treatment on Improving the Performance of Solar Cell N-ITO/p-Si Junction: Structural, Optical, and Electrical Characterizations. Mater. Today Commun..

[39] Sofi A.H., Shah M.A., Asokan K. (2018). Structural, Optical and Electrical Properties of ITO Thin Films. J. Electron. Mater..

[40] Manzano C.V., Rojas A.A., Decepida M., Abad B., Feliz Y., Caballero-Calero O., Borca-Tasciuc D.-A., Martin-Gonzalez M. (2013). Thermoelectric Properties of Bi_2_Te_3_ Films by Constant and Pulsed Electrodeposition. J. Solid State Electrochem..

[41] Nisha M., Anusha S., Antony A., Manoj R., Jayaraj M.K. (2005). Effect of Substrate Temperature on the Growth of ITO Thin Films. Appl. Surf. Sci..

[42] Tauc J. (1968). Optical Properties and Electronic Structure of Amorphous Ge and Si. Mater. Res. Bull..

[43] Hamberg I., Granqvist C.G. (1986). Evaporated Sn-doped In_2_O_3_ Films: Basic Optical Properties and Applications to Energy-efficient Windows. J. Appl. Phys..

[44] Park H.-K., Jeong J.-A., Park Y.-S., Kim H.-K., Cho W.-J. (2009). Electrical, Optical, and Structural Properties of InZnSnO Electrode Films Grown by Unbalanced Radio Frequency Magnetron Sputtering. Thin Solid Films.

[45] Akhmedov A.K., Abduev A.K., Murliev E.K., Belyaev V.V., Asvarov A.S. (2023). Transparent Conducting Amorphous IZO Thin Films: An Approach to Improve the Transparent Electrode Quality. Materials.

[46] Ali A.H., Shuhaimi A., Hassan Z. (2014). Structural, Optical and Electrical Characterization of ITO, ITO/Ag and ITO/Ni Transparent Conductive Electrodes. Appl. Surf. Sci..

[47] Zhu B.L., Ma J.M., Lv K., Wang C.J., Wu J., Gan Z.H., Liu J., Shi X.W. (2020). Improvement of Transparent Conductive Properties of GZO/Cu/GZO Tri-Layer Films by Introducing H_2_ into Sputtering Atmosphere. Superlattices Microstruct..

[48] Lee S.Y., Park Y.S., Seong T.-Y. (2019). Optimized ITO/Ag/ITO Multilayers as a Current Spreading Layer to Enhance the Light Output of Ultraviolet Light-Emitting Diodes. J. Alloys Compd..

[49] Haacke G. (1976). New Figure of Merit for Transparent Conductors. J. Appl. Phys..

[50] Ren H., Zou X., Cheng J., Ling T., Bai X., Chen D. (2018). Facile Solution Spin-Coating SnO_2_ Thin Film Covering Cracks of TiO_2_ Hole Blocking Layer for Perovskite Solar Cells. Coatings.

[51] Satpute S.D., Bhujbal P.K., Shaikh S.F., Patil S.A., Jadkar S.R., More S.A. (2023). TiO_2_ Blocking Layer Incorporated TiO_2_/In_2_O_3_-Based Photoanode for DSSC Application. J. Mater. Sci. Mater. Electron..

[52] Chaudhary D.K., Kumar P., Kumar L. (2017). Realization of Efficient Perovskite Solar Cells with MEH: PPV Hole Transport Layer. J. Mater. Sci. Mater. Electron..

[53] Oku T. (2020). Crystal Structures of Perovskite Halide Compounds Used for Solar Cells. Rev. Adv. Mater. Sci..

[54] Sewvandi G.A., Kodera K., Ma H., Nakanishi S., Feng Q. (2016). Antiferroelectric Nature of CH_3_NH_3_PbI_3_−XClx Perovskite and Its Implication for Charge Separation in Perovskite Solar Cells. Sci. Rep..

[55] Song W., Rakocevic L., Thiruvallur Eachambadi R., Qiu W., Bastos J.P., Gehlhaar R., Kuang Y., Hadipour A., Aernouts T., Poortmans J. (2021). Improving the Morphology Stability of Spiro-OMeTAD Films for Enhanced Thermal Stability of Perovskite Solar Cells. ACS Appl. Mater. Interfaces.

